# Effects of nonsynonymous single nucleotide polymorphisms of the *KIAA1217*, *SNTA1* and *LTBP1* genes on the growth traits of Ujumqin sheep

**DOI:** 10.3389/fvets.2024.1382897

**Published:** 2024-05-02

**Authors:** Zhichen Liu, Qing Qin, Chongyan Zhang, Xiaolong Xu, Dongliang Dai, Mingxi Lan, Yichuan Wang, Jingwen Zhang, Dan Zhao, Deqing Kong, Tian Qin, Danni Wu, Xuedan Gong, Xingyu Zhou, Alatan Suhe, Zhixin Wang, Zhihong Liu

**Affiliations:** ^1^College of Animal Science, Inner Mongolia Agricultural University, Hohhot, China; ^2^East Ujumqin Banner Hersig Animal Husbandry Development Limited Liability Company, Xilin Gol League, Xilinhot, China; ^3^Key Laboratory of Animal Genetics, Breeding and Reproduction, Inner Mongolia Agricultural University, Hohhot, China; ^4^Key Laboratory of Mutton Sheep Genetics and Breeding, Ministry of Agriculture, Hohhot, China; ^5^Goat Genetics and Breeding Engineering Technology Research Center, Hohhot, China

**Keywords:** Ujumqin sheep, nsSNP, *KIAA1217*, *SNTA1*, *LTBP1*

## Abstract

Sheep body size can directly reflect the growth rates and fattening rates of sheep and is also an important index for measuring the growth performance of meat sheep. In this study, high-resolution resequencing data from four sheep breeds (Dorper sheep, Suffolk sheep, Ouessant sheep, and Shetland sheep) were analyzed. The nonsynonymous single nucleotide polymorphisms of three candidate genes (*KIAA1217*, *SNTA1*, and *LTBP1*) were also genotyped in 642 healthy Ujumqin sheep using MALDI-TOFMS and the genotyping results were associated with growth traits. The results showed that different genotypes of the *KIAA1217* g.24429511T>C locus had significant effects on the chest circumferences of Ujumqin sheep. The *SNTA1* g.62222626C>A locus had different effects on the chest depths, shoulder widths and rump widths of Ujumqin sheep. This study showed that these two sites can be used for marker-assisted selection, which will be beneficial for future precision molecular breeding.

## 1 Introduction

Sheep (*Ovis aries*) are economically one of the most important animals in the world (1), providing humans with a stable and high-quality source of animal protein and animal products such as skin, hair, and milk. Since sheep were domesticated (2), they have inhabited all parts of the world following the migration of humans. In this process, both the natural environment and artificial selection have had a profound impact on the domestication of sheep, resulting in large differences in the appearances, phenotypes, and morphological structures of sheep in different regions. For example, Dorper sheep native to South Africa and Suffolk sheep (3, 4) from the United Kingdom are famous for their fast growth rates and high meat production. The average adult sheep weight range can reach 85–120 kg. In sharp contrast, small sheep, such as Ouessant sheep and Shetland sheep (5, 6), have average adult body weights ranging from 45 to 75 kg. The growth traits of sheep (such as weight, height and length) can directly reflect the growth rates and fat growth rates of sheep and are also an important indicator of the growth performance of sheep. Therefore, understanding sheep growth traits is highly important for livestock production. At present, the common sheep varieties in China include Ujumqin sheep, Hu sheep, Xiaowei Han sheep, Tibetan sheep and others (7–10). As an excellent local variety differentiated from Mongolian sheep, Ujumqin sheep gradually developed after long-term selection and breeding and are well known for their fast growth rate and delicious meat.

In recent years, following the rapid development of biotechnology and genomics, molecular marker technology has improved. Single-nucleotide polymorphisms (SNPs) are third-generation molecular markers after restricted fragment length polymorphisms (RFLPs) and microsatellite polymorphisms (MPPs) (11). SNPs refer to DNA sequence polymorphisms caused by single nucleotide variations in the genome, including single base insertions, deletions, transitions, and transversions (12). SNPs can be classified into nonsynonymous single nucleotide polymorphisms (nsSNPs) and synonymous single nucleotide polymorphisms (sSNPs) according to their effects on gene transcription and protein translation. Nonsynonymous single nucleotide polymorphisms represent common genetic variants that alter the translated amino acid sequence, and this may affect the structure or function of the expressed protein (13). This genetic variation may lead to alterations in individual traits, such as body length and height growth traits. In this context, the results of an increasing number of studies have shown that nsSNPs have important effects on the growth traits of sheep (14, 15). By continuously mining and verifying major gene loci related to sheep economic traits, including the *MSTN* gene affecting sheep muscle growth and development, the *TBXT* gene affecting sheep tail type, and the *FGF5* candidate gene related to wool length, researchers have applied these polymorphisms of major genes in production practice through gene editing and other technologies. This approach can greatly increase the economic value of sheep; for example, biallelic knockout of *MSTN* expression in sheep was successfully achieved using CRISPR/Cas9 gene editing. Compared with wild-type (WT) sheep, *MSTN* knockout sheep have greater muscle mass and muscle fiber diameters, but their meat quality and taste are not affected (16). CRISPR/Cas9-mediated deletion of the *FGF5* gene not only results in the loss of its activity but also promotes the growth of sheep wool and cashmere, thereby increasing length and yield (17). These achievements have brought new optimism and opportunities for the development of animal husbandry. Nevertheless, the major gene loci involved in sheep growth traits still require research, especially with the deepening of our understanding of the sheep genome driven by biotechnology. There are still some potentially important gene loci that have not been revealed. Therefore, it is of great theoretical and practical significance to identify and verify the relevant sheep growth trait sites; it is expected that this investigation will provide a breakthrough for improving sheep production performance and economic benefits. It is important to identify potential genetic markers that affect the growth traits or reproductive characteristics of sheep and to accelerate the sheep breeding process.

In this study, resequencing data from four sheep breeds were divided into high-yield and low-yield groups according to body size, and population differentiation index (FST) analysis was used to identify genes and mutation sites related to the body size of the sheep. Based on the FST results, we performed GO and KEGG enrichment analyses for the top 5% of the candidate genes. Twenty-four nsSNPs across three candidate genes, *KIAA1217*, *STNA1* and *LTBP1*, were identified. However, whether these nsSNPs have an effect in Ujumqin sheep is unclear. Therefore, we collected blood from 642 Ujumqin sheep for genotyping of the above sites, matched the genotyping results with body size data, and verified the molecular markers of the main effect growth trait sites in the genomes of Ujumqin sheep.

## 2 Materials and methods

### 2.1 Sample collection

The experimental animals used in this study were bred in their country of origin and were not crossed with other sheep breeds. A total of 642 Ujumqin sheep (260 males +382 females) were obtained from the East Ujumqin Banner breeding farm in Xilin Gol League, Inner Mongolia. All healthy animals were fed freely under the same natural conditions. Venous blood collection was performed on sheep using a vacuum blood collection vessel, and the sample storage conditions were −20°C. The body size data of the sheep were measured manually. For detailed measurement methods, see a previously published article (18).

### 2.2 Data sources

The raw data for this study were downloaded from the BioProject database of NCBI (PRJNA624020). The dataset contained resequencing data for four breeds of sheep (Dorper sheep, Suffolk sheep, Ouessant sheep and Shetland sheep), for a total of 37 samples.

### 2.3 Sequence read mapping and SNP calling

Approximately 82.86 Gb of the original sequence was obtained from each sample, with an average depth of 27.4× covering the clean reads (Supplementary Datas 1, 2). Using default parameters, the 150 bp counterpart reads were mapped to the sheep reference genome Oar v.4.0 using the Burrows–Wheeler Aligner (19). The mapping results were then converted to BAM format and sorted using SAMtools (20). After mapping, we made SNP calls to all individuals using the Bayesian method implemented in SAMtools and the Genome Analysis Toolkit (GATK) (21). Then, SnpEff software (22) was used to annotate the structures of the mutation sites.

### 2.4 FST analysis

The four breeds of sheep were divided into two groups according to their body sizes (high-yield group: Dorper sheep, Suffolk sheep; low-yield group: Ouessant sheep, Shetland sheep). The FST value was estimated using a 100 kb sliding window on each chromosome with a window step size of 10 kb. The average FST values are the values for the entire genomes of different populations. When the FST value of the sliding window was greater than 95% of the FST value of the genome range, it was selected as a significant window. The overlapping significant windows were then merged into segments that were considered highly differentiated regions between populations. Genes within these regions of differentiation are considered candidates for selective elimination. We used the VCFtools 0.1.16 software package (23) for data collection for the FST calculation method: (1) Calculation of in-population genetic variation: For each genetic marker, the allele frequency of each population was calculated. The degree of heterozygosity (Ho) or allelic diversity within each population was calculated. (2) Calculation of interpopulation genetic variation: The average allele frequency of all populations was calculated. These mean frequencies were used to calculate the expected heterozygosity (Ht) of the population. (3) Calculation of FST:


$$FST=\frac{\left(Ht-Hs\right)}{Ht}$$

Here, Ht is the overall expected heterozygosity between populations, and Hs is the weighted average expected heterozygosity within populations.

### 2.5 Enrichment analysis of key genes

Gene Ontology (GO) and Kyoto Encyclopedia of Genes and Genomes (KEGG) enrichment analyses were performed for the top 5% of the FST genes. GO enrichment analysis was performed using the online tool g: Profiler (24), and GO classifications included biological processes (BP), cellular components (CC), and molecular functions (MF). The significantly enriched GO items were selected according to *p* < 0.05. KOBAS 3.0 (25) was used for KEGG pathway enrichment analysis, and *p* < 0.05 was considered the screening criterion for significant enrichment.

### 2.6 Design and synthesis of primers and classification of SNPs

Based on the FST analysis results of this study and referring to previous results (26), primer design was performed using the upstream and downstream 200 bp sequence information of nsSNPs of common genes significantly selected by XP-CLR in the top 5% of FSTs. The primers were designed using the Agena online software Design Suite 2.0.^1^ The designed primer sequence was derived and then synthesized by Invitrogen. The mutation sites were identified by stroma-assisted laser desorption ionization time-of-flight mass spectrometry (MALDI-TOF-MS). The genotyping system used was the MassARRAY® MALDI-TOF system developed by Agena.

### 2.7 Statistical analysis

The phenotypic data of Ujumqin sheep were statistically analyzed using Excel 2021 software. SPSS 22.0 software (27) was used to analyze the correlation between genotypes and phenotypic traits. *P* < 0.05 indicated significant differences, and *p* < 0.01 indicated extremely significant differences. The following general linear model (GLM) was used for analysis: Y = μ + G + m + e.

Y, trait measurement value; μ, population mean; G, genotype effect; m, sex effect; e, random residual.

### 2.8 nsSNP protein structure prediction

TBtools (28) was used to extract CDS information before and after mutation based on reference genome information. RNAfold^2^ online software was used to predict the secondary structure of mutated mRNAs. SOPMA (29) was used to predict protein secondary structure. After the amino acid sequence using CDS sequence can be converted to SWISS-MODEL^3^ online software to forecast the mutant protein tertiary structure before and after.

## 3 Results

### 3.1 Selection of candidate genes for sheep body size

**Figure 1:**
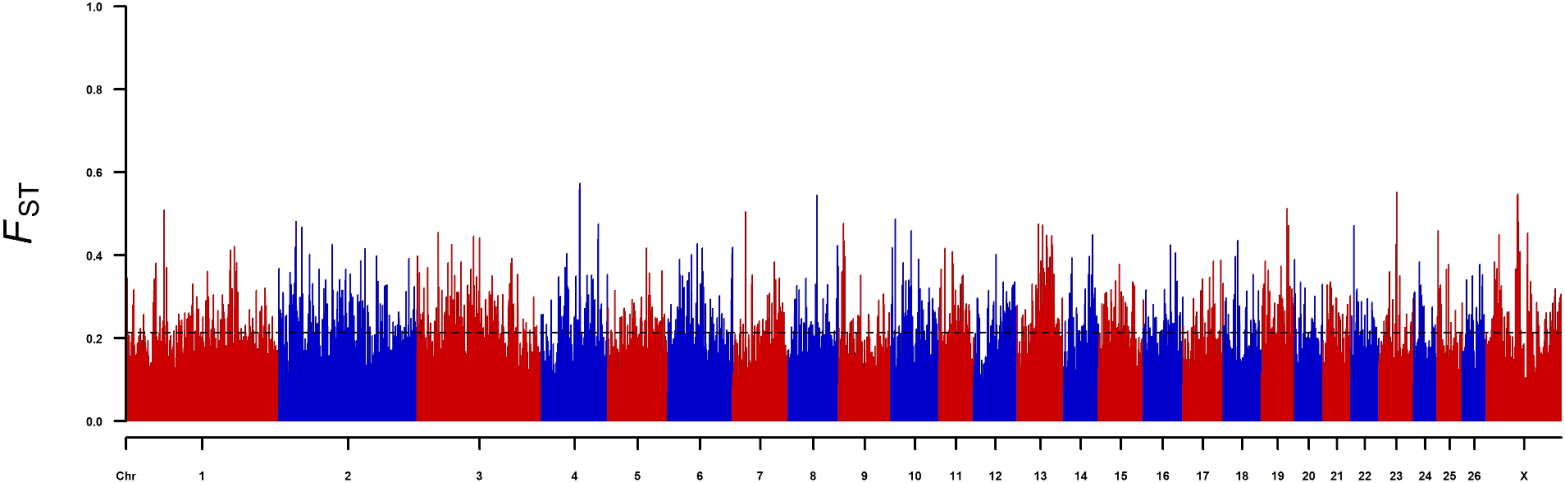
Genome-wide distribution of FST in the high-yield and low-yield groups; the *X*-axis represents chromosomes, the *Y*-axis represents FST values, and the black dashed line represents the significance threshold.

To identify candidate genes that affect sheep body size, we conducted FST selection signal analysis on the high-yield and low-yield groups of sheep (Figure 1), and a total of 1747 significant genes were selected as the top 5% of the FST. Analysis of these 1747 genes revealed that three genes (*KIAA1217*, *SNTA1* and *LTBP1*) were significantly selected by the XP-CLR method used in previous studies. Therefore, we selected nsSNPs of the above three genes for further study (Table 1).

**Table 1:** The nonsynonymous single nucleotide polymorphism sites in the *KIAA1217*, *SNTA1*, and *LTBP1* genes.

SNP	Gene name	Mutation type	Position	REF	ALT
g.24086608A > C	*KIAA1217*	Missense variant	Chr13: 24086608	A	C
g.24125201C > T	*KIAA1217*	Missense variant	Chr13: 24125201	C	T
g.24125227G > A	*KIAA1217*	Missense variant	Chr13: 24125227	G	A
g.24125228T > C	*KIAA1217*	Missense variant	Chr13: 24125228	T	C
g.24125285G > A	*KIAA1217*	Missense variant	Chr13: 24125285	G	A
g.24256607C > T	*KIAA1217*	Stop gained	Chr13: 24256607	C	T
g.24256993C > T	*KIAA1217*	Missense variant	Chr13: 24256993	C	T
g.24351854A > G	*KIAA1217*	Missense variant	Chr13: 24351854	A	G
g.24387021G > A	*KIAA1217*	Missense variant	Chr13: 24387021	G	A
g.24392399A > G	*KIAA1217*	Missense variant	Chr13: 24392399	A	G
g.24399197C > A	*KIAA1217*	Missense variant	Chr13: 24399197	C	A
g.24406459C > T	*KIAA1217*	Missense variant	Chr13: 24406459	C	T
g.24424378C > T	*KIAA1217*	Missense variant	Chr13: 24424378	C	T
g.24428968T > C	*KIAA1217*	Missense variant	Chr13: 24428968	T	C
g.24429511T > C	*KIAA1217*	Missense variant	Chr13: 24429511	T	C
g.24429581T > A	*KIAA1217*	Missense variant	Chr13: 24429581	T	A
g.62220063G > A	*SNTA1*	Missense variant	Chr13: 62220063	G	A
g.62222626C > A	*SNTA1*	Missense variant	Chr13: 62222626	C	A
g.62247870T > C	*SNTA1*	Missense variant	Chr13: 62247870	T	C
g.90345529C > T	*LTBP1*	Missense variant	Chr3: 90345529	C	T
g.90377522G > A	*LTBP1*	Missense variant	Chr3: 90377522	G	A
g.90398299A > C	*LTBP1*	Missense variant	Chr3: 90398299	A	C
g.90427996G > T	*LTBP1*	Missense variant	Chr3: 90427996	G	T
g.90642790G > C	*LTBP1*	Missense variant	Chr3: 90642790	G	C

### 3.2 Enrichment analysis of candidate genes

**Figure 2:**
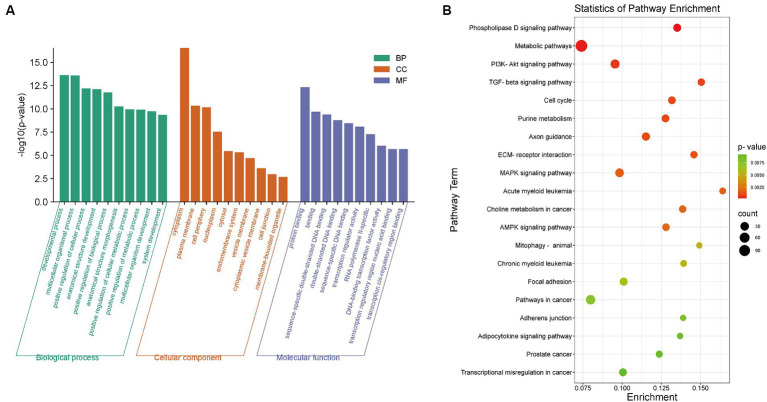
The results of GO/KEGG enrichment analysis of the top 5% of genes in the FST selection signal. **(A)** The results of GO enrichment analysis. Green, orange and blue represent biological processes, cell components and molecular functions in GO enrichment terms, respectively. **(B)** The results of KEGG enrichment analysis. The *X*-axis represents the ratio of differentially enriched genes to the total gene number of this term, and the *Y*-axis represents the enriched function/pathway. The color represents the degree of enrichment significance, and the closer to red the color is, the more significant the enrichment is. The size of the circle represents the number of genes enriched.

To investigate which GO enrichment terms and signaling pathways were associated with the top 5% of genes significantly enriched in the FST, we conducted GO and KEGG enrichment analyses of these 1747 genes (Figure 2). According to the GO classification statistics, 214 terms were grouped into three GO categories: cellular components, molecular functions, and biological processes. Among the three GO categories, the most significantly enriched were cytoplasm, protein binding and developmental process. GO enrichment analysis revealed that most of these genes were involved to varying degrees in the development of organisms (Figure 2A).

In addition, we performed KEGG pathway enrichment analysis to explore the most active pathways related to these genes. The results showed that most of the enriched pathways were related to biological development, such as the phospholipase D signaling pathway, the PI3K/Akt signaling pathway, the TGF-β signaling pathway, and cell cycle (Figure 2B).

### 3.3 Correlations between growth traits and nsSNPs in Ujumqin sheep

**Figure 3:**
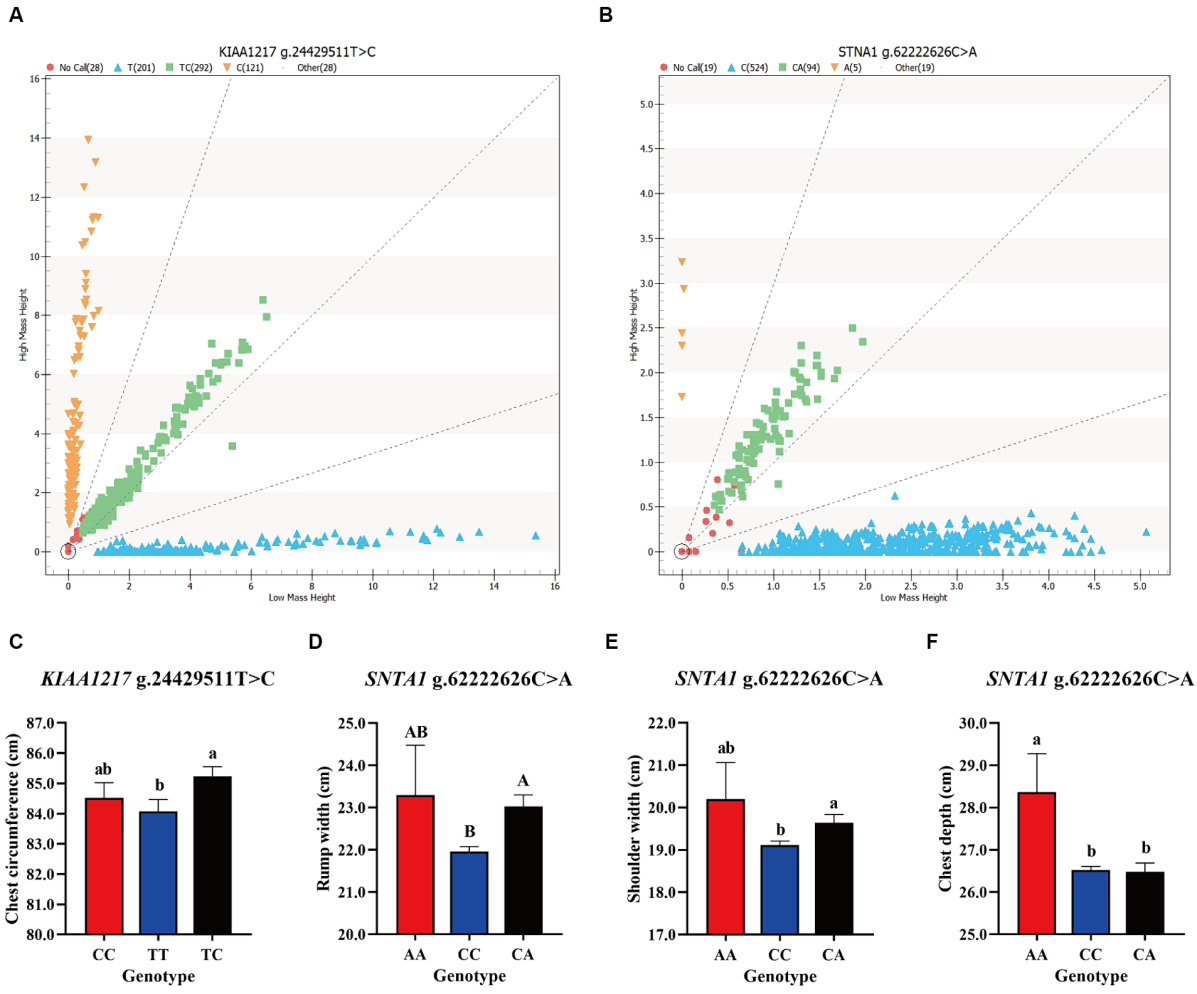
Results of site genotyping and association analysis; **(A,B)** show the genotyping results of *KIAA1217* g.24429511T > C and *SNTA1* g.62222626C > A in the Ujumqin sheep population, respectively. The genotypes near the *X*-axis were of low molecular weight, and those near the *Y*-axis were of high molecular weight. Blue and orange represent pure genotypes, green represents heterozygous genotypes, and red represents undetected samples. **(C–F)** Show the effects of different genotypes of two SNP loci on Ujumqin sheep production traits. **(C)** The chest sizes of sheep with the TC genotype at *KIAA1217* g.24429511T > C were significantly greater than those of sheep with the TT genotype. At the *SNTA1* g.62222626C > A locus, sheep with the **(D)** CA genotype were significantly wider than sheep with the CC genotype, **(E)** sheep with the CA genotype were significantly wider than those with the CC genotype, and **(F)** sheep with the AA genotype were significantly deeper than those with the CC and CA genotypes. Different letters on the shoulder of the same column of data indicate statistically significant differences, lowercase letters represent significant differences (*p* < 0.05), and uppercase letters represent extremely significant differences (*p* < 0.01).

The DNA of 642 Ujumqin sheep was genotyped and the next step was to analyze the loci with more than 95% detection rate. Among the 24 nsSNPs, only 2 met the above conditions, namely, *KIAA1217* g.24429511T > C and *SNTA1* g.62222626C > A (Figures 3A,B). Analysis of the genotype frequencies and allele frequencies of these two loci revealed 3 genotypes at each locus. The TC genotype in the *KIAA1217* g.24429511T > C locus was the dominant genotype, and the genotype frequency was 0.48, indicating moderate polymorphism (0.25 < PIC<0.5). At the *SNTA1* g.62222626C > A locus, the CC genotype was the dominant genotype, the genotype frequency was 0.84, and the polymorphism frequency was low (PIC<0.25). In addition, according to the χ2 test, both of the above two SNPs reached HWE (*p* > 0.05) (Table 2). The results of the association analysis between the typing results of the two loci and the 6-month-old body size data of Ujumqin sheep are shown in Table 3. The presence of different genotypes at the *KIAA1217* g.24429511T > C locus had significant effects on the chest circumference of Ujumqin sheep (Figure 3C), and the chest circumference of individuals with the TC genotype was significantly greater than that of individuals with the TT genotype (*p* < 0.05). The chest depths of Ujumqin sheep with an AA genotype at the *SNTA1* g.62222626C > A locus were greater than those of CC genotype and CA genotype sheep (Figure 3F), and the difference was significant (*p* < 0.05). The shoulder widths of CA genotype sheep were significantly greater than those of CC genotype sheep (Figure 3E) (*p* < 0.05), and the differences in rump width were highly significant (*p* < 0.01), as shown in Figure 3D.

**Table 2:** Genetic parameters of nsSNPs in the experimental population of Ujumqin sheep.

SNP	Genotype	Genotype frequency	Allele	Allele frequency	He	Ho	PIC	HWE (χ^2^)
*KIAA1217* g.24429511T > C	CC (121)	0.20	T	0.57	0.48	0.52	0.35	0.62
	TT (201)	0.33	C	0.43				
	TC (292)	0.48						
*SNTA1* g.62222626C > A	AA (5)	0.01	C	0.92	0.15	0.85	0.14	0.04
	CC (524)	0.84	A	0.08				
	CA (94)	0.15						

**Table 3:** Genetic parameters of nsSNPs in the experimental population of Ujumqin sheep.

SNP	Genotype	Body slanting length	Height at wither	Hip height	Chest depth	Shoulder width	Rump width	Chest circumference
*KIAA1217* g.24429511T > C	CC (121)	63.77 ± 0.38	59.95 ± 0.36	62.18 ± 0.38	26.61 ± 0.19	18.96 ± 1.80	22.05 ± 0.24	84.52 ± 0.51^ab^
	TT (201)	64.27 ± 0.29	59.86 ± 0.28	61.50 ± 0.30	26.45 ± 0.15	19.23 ± 0.14	21.97 ± 0.19	84.08 ± 0.39^b^
	TC (292)	63.56 ± 0.24	59.87 ± 0.23	61.97 ± 0.25	26.55 ± 0.12	19.300.11	22.24 ± 0.16	85.23 ± 0.33^a^
*SNTA1* g.62222626C > A	AA (5)	65.24 ± 1.85	60.62 ± 1.78	62.30 ± 1.86	28.37 ± 0.91^a^	20.20 ± 0.86^ab^	23.30 ± 1.18^AB^	84.36 ± 2.25
	CC (524)	63.84 ± 0.18	59.85 ± 0.18	61.98 ± 0.19	26.52 ± 0.09^b^	19.12 ± 0.09^b^	21.96 ± 0.12^B^	84.73 ± 0.25
	CA (94)	63.97 ± 0.43	60.26 ± 0.41	62.32 ± 0.43	26.48 ± 0.21^b^	19.64 ± 0.20^a^	23.03 ± 0.27^A^	84.69 ± 0.57

Different uppercase letters in the same column represent very significant differences (*p* < 0.01), different lowercase letters represent significant differences (*p* < 0.05), and the same letters represent no significant differences.

### 3.4 Prediction of nsSNP protein structure related to growth traits of Ujumqin sheep

**Figure 4:**
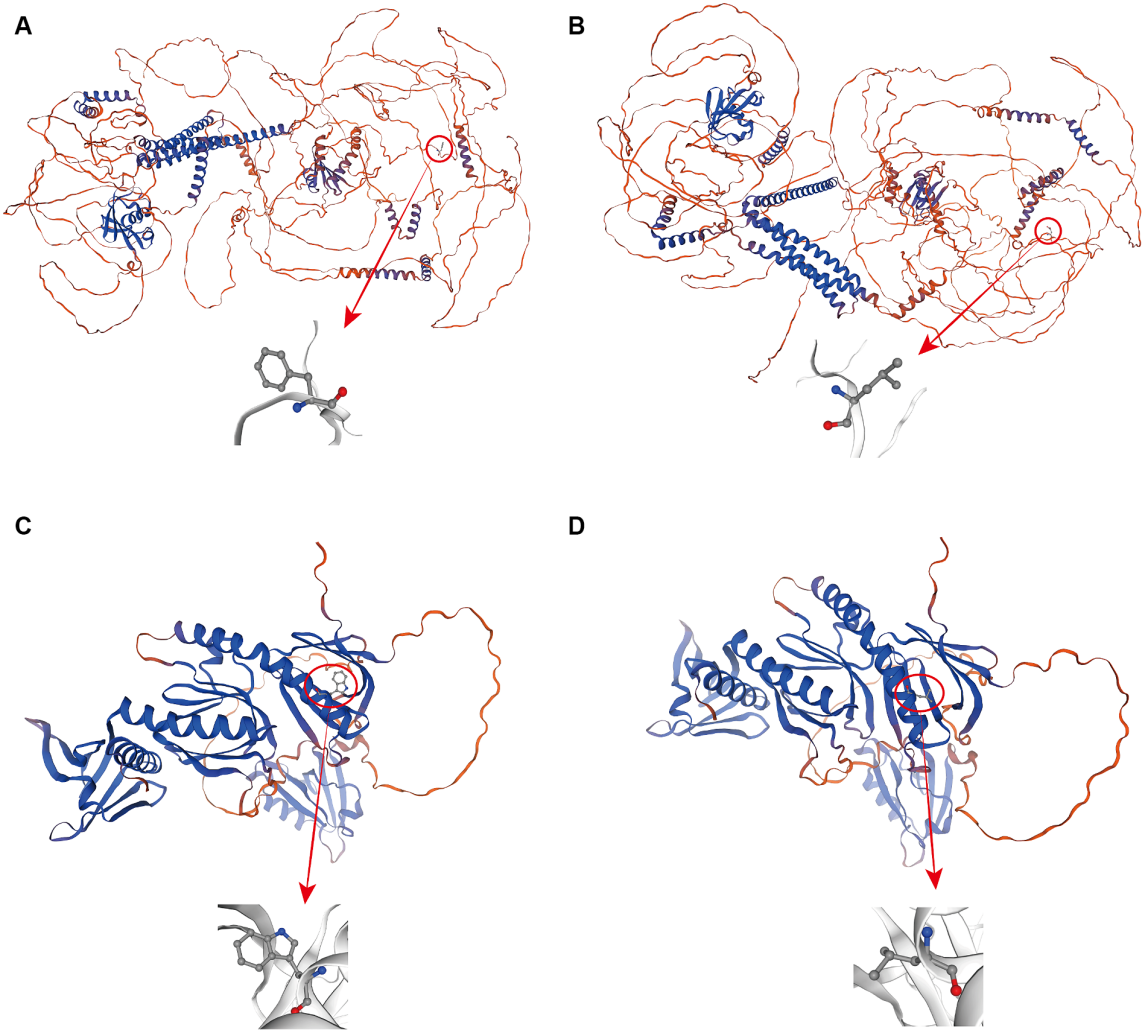
Protein tertiary structure prediction map before and after mutation at 2 mutation sites; **(A–D)** represent the wildtype and mutant proteins encoded by *KIAA1217* and the wildtype and mutant proteins encoded by *SNTA1*, respectively.

To better understand how the above two mutation sites affect the expression of genes involved in the growth trait changes of Ujumqin sheep, we used TBtools to extract the CDSs of genes before and after the mutation and used online software to predict the secondary structures of mRNA and the secondary structures and tertiary structures of encoded proteins. The results showed that mutations at both sites resulted in changes in the original amino acid sequence, and mutations at *KIAA1217* g.24429511T > C resulted in a change in amino acid 1,493 from the original phenylalanine to leucine (Figures 4A,B). Amino acid 260 at *SNTA1* g.62222626C > A changed from tryptophan to leucine (Figures 4C,D). Secondary structure analysis of mRNA before and after mutation revealed that the minimum structural free energy of mRNA before and after the *KIAA1217* g.24429511T > C mutation changed from −1983.90 kcal/mol to −1984.40 kcal/mol. The minimum structural free energy of the mRNA before and after the *SNTA1* g.62222626C > A site changed from −692.50 kcal/mol to −695.50 kcal/mol, and the stability increased (Supplementary Figure 1). By analyzing the changes in amino acids at the mutation sites and their effects on protein secondary structures, it was found that the proportion of α-helix and random coils increased after mutation at *KIAA1217* g.24429511T > C, and the proportion of β-turn and extended strands decreased. There was no change in the proportion of secondary structures after the *SNTA1* g.62222626C > A mutation (Table 4).

**Table 4:** Results of protein secondary structure prediction.

Gene		α-helix	β-turn	Extended strand	Random coil
*KIAA1217*	Wild type	25.21%	2.95%	9.68%	62.16%
	Mutant	25.41%	2.85%	9.52%	62.22%
*SNTA1*	Wild type	29.11%	5.74%	21.98%	43.17%
	Mutant	29.11%	5.74%	21.98%	43.17%

## 4 Discussion

Against the background of natural selection, some selective intervention behaviors of humans may cause mutations in some sites of the animal body so that the genetic traits of the organism are diverse. Phenotypic alterations are often caused by functional mutations in genes that control the trait, so such mutations can serve as valid molecular markers for marker-assisted breeding (30). Among these mutations, missense mutations are considered to affect the body phenotype by affecting the mRNA expression of the related genes and the structures of the proteins. In this study, the g.24429511T > C and g.62222626C > A mutations, which are missense mutations that cause differences in growth traits, were located in exon 19 and exon 4 of the gene, respectively. Although both sites lead to changes in amino acids encoded, there are some differences between the two mutations. The g.24429511T > C missense mutation caused the encoded amino acid to change from phenylalanine to leucine, and the amino acid properties changed from polar to nonpolar after the mutation, which may have led to changes in protein properties. Further analysis revealed that mutation of this site resulted in a decrease in the minimum structural free energy of the mRNA secondary structure, an increase in stability, an increase in the proportion of α-helix and random coils, and a decrease in the proportion of β-turn and extended strands. After the missense mutation at g.62222626C > A, the encoded amino acid changed from tryptophan to leucine and from a polar amino acid to a nonpolar amino acid. However, from the perspective of protein secondary structure, the mutation at this site did not cause changes in the proportions of α-helix, extended strands, β-turn or random coils. The main reason may be that amino acids form extended strands in the secondary structure before and after mutation. The *SNTA1* gene encodes a cytoplasmic peripheral membrane scaffold protein. In terms of tertiary structure, changes in the amino acids of the protein may affect the structure of the protein, thus affecting the binding of related proteins and leading to the emergence of different phenotypes.

*KIAA1217* (a sickle tail protein homolog) has a curly helix region and an actin interaction domain. The proteins it encodes are necessary for normal disc development, dendritic spine morphogenetic regulation, embryonic skeletal system development by regulating cell migration, multicellular biological development, and substrate adhesion-dependent cell proliferation. Mutations in *KIAA1217* are associated with malformations in the human backbone and tail vertebrae, and in mice, they affect the development of the spine, resulting in a reduced number of tail vertebrae and a characteristic short tail (31). Recently, researchers have shown that rapid evolution of the regulatory region of this gene in apes may lead to tail loss, which may be related to mutations in specific gene regulatory sequences (32). It has been confirmed that the vertebrae of sheep and humans are most similar in the thoracic and lumbar regions, although they show substantial differences in some dimensions (33). *SNTA1* encodes the α1-synthetic protein, a scaffold protein that is a component of the anti-muscular dystrophin-associated protein complex (34). *SNTA1* is the link between the extracellular matrix, the intracellular signaling apparatus, and the actin cytoskeleton. *SNTA1* is involved in the regulation of the actin cytoskeleton and actin recombination (35). The *SNTA1* signaling axis plays an important role in cytoskeletal tissue (36), and researchers have found that the *SNTA1* gene is associated with amino acid and ion channel binding in different parts of bovine muscle (37). The sheep *LTBP1* gene, located on chromosome 3, encodes TGF-β-binding protein 1 and is a member of the potential TGF-β-binding protein family. One indel of the *LTBP1* gene was detected in four sheep breeds, and the effect of the *LTBP1* gene on the growth traits of small-tailed cold sheep may be related to sex (38). Cao et al. conducted genome-wide DNA methylation sequencing in a subpopulation of Chinese Mongolian sheep and found that DNA methylation in three regions and two CpG sites in *LTBP1* was significantly correlated with its RNA expression, and this gene was also identified as a potential candidate gene associated with weight variation (39). Unfortunately, 5 nsSNPSs of the *LTBP1* gene were not detected in the population genotyping results of Ujumqin sheep in this study. Moreover, exploring the influence of the *LTBP1* gene on the growth traits of Ujumqin sheep will also constitute a direction of follow-up research.

In recent years, following the development of biotechnology and genome research, an increasing number of SNPs related to the growth traits of livestock and poultry have been discovered. Studies have shown that the g.3148C > T polymorphism of the *SIRT1* gene affects the heart circumference of Tibetan sheep, and the g.8074 T > A polymorphism of the *SIRT2* gene is significantly correlated with body weight and body length (40). Cao et al. discovered OARX_76354330.1 and s64890.1 to be functional SNPs for growth traits of Hu sheep through genome-wide association analysis of body weight and identified three candidate genes related to body weight in Hu sheep (41): *CAPN6*, *ITGA11* and *SCMH1*. By using genome-wide high-density SNP data (600 K) for selective scanning tests for important phenotypic traits, researchers have identified genes related to sheep body size, such as *RMI1* and *SCD5* (42). In the selection of sheep genotypes, it is necessary to comprehensively select SNP genotypes corresponding to the target traits; we therefore need to use different methods to identify additional SNP sites that affect sheep growth traits. Overall, this study used resequencing data from different sheep breeds to mine candidate genes and nsSNPs affecting sheep body size and verified their roles in Ujumqin sheep, thereby obtaining two molecular markers that could be applied in production practice. However, it is not yet clear whether these sites are equally useful in other sheep breeds, and this will be an important direction for our future research. Our results provide new insights into the mining of SNPs related to sheep growth and provide new genetic markers for the genetic improvement in Ujumqin sheep.

## 5 Conclusion

In this study, resequencing data from four sheep breeds were used to identify three genes related to the body size of sheep. Twenty-four nsSNPs across three genes were identified in Ujumqin sheep. Association analysis revealed that different genotypes of two SNP loci had differences in chest circumference, chest depth, body width and caudal breadth, and mRNA secondary structure stability was enhanced after mutation. These SNPs can be used as a molecular marker for Ujumqin sheep breeding and lay a foundation for future precise molecular breeding.

## Data availability statement

The data used in this study is sourced from the BioProject database of NCBI, numbered PRJNA624020.

## Ethics statement

The animal study was approved by in this study, blood was collected in accordance with the International Guiding Principles for Biomedical Research involving animals and approved by the Special Committee on Scientific Research and Academic Ethics of Inner Mongolia Agricultural University, responsible for the approval of Biomedical Research Ethics of Inner Mongolia Agricultural University [Approval No. (2020) 056]. No specific permissions were required for these activities, and no endangered or protected species were involved. The study was conducted in accordance with the local legislation and institutional requirements.

## Author contributions

ZCL: Writing – original draft, Writing – review & editing. QQ: Writing – original draft, Writing – review & editing. CZ: Writing – original draft, Writing – review & editing. XX: Writing – original draft, Writing – review & editing. DD: Writing – original draft, Writing – review & editing. ML: Writing – original draft, Writing – review & editing. YW: Writing – original draft, Writing – review & editing. JZ: Writing – original draft, Writing – review & editing. DZ: Writing – original draft, Writing – review & editing. DK: Writing – original draft, Writing – review & editing. TQ: Writing – original draft, Writing – review & editing. DW: Writing – original draft, Writing – review & editing. XG: Writing – original draft, Writing – review & editing. XZ: Writing – original draft, Writing – review & editing. AS: Writing – original draft, Writing – review & editing. ZW: Writing – original draft, Writing – review & editing. ZHL: Writing – original draft, Writing – review & editing.

## Footnotes

^1^ https://support.agenabio.com/s/online-tools

^2^ http://rna.tbi.univie.ac.at/

^3^ https://swissmodel.expasy.org/
