# Supplementary material for: Effects of nonsynonymous single nucleotide polymorphisms of the KIAA1217, SNTA1 and LTBP1 genes on the growth traits of Ujumqin sheep
Source: Front Vet Sci. 2024 May 2;11:1382897. doi: 10.3389/fvets.2024.1382897 (PMC11097667; doi:10.3389/fvets.2024.1382897)
Supplement: Supplementary file 3 [file Data_Sheet_3.DOCX]

Supplementary Material

Effects of non-synonymous mutation single nucleotide polymorphisms of KIAA1217, SNTA1 and LTBP1 genes on growth traits of Ujumqin sheep

Zhichen Liu, Qing Qin, Chongyan Zhang, Xiaolong Xu, Dongliang Dai, Mingxi Lan, Yichuan Wang, Jingwen Zhang, Dan Zhao, Deqing Kong, Tian Qin, Danni Wu, Xuedan Gong, Xingyu Zhou, Alatan Suhe, Zhixin Wang and Zhihong Liu*

*** Correspondence:** Corresponding Author: liuzh7799@163.com

# Supplementary Data

Supplementary Data 1 is the statistical data of 37 resequenced samples and Supplementary Data 2 is the comparison between each sample and the reference genome. Supplementary Data is submitted in EXCEL format.

# Supplementary Figures and Tables

## Supplementary Figures


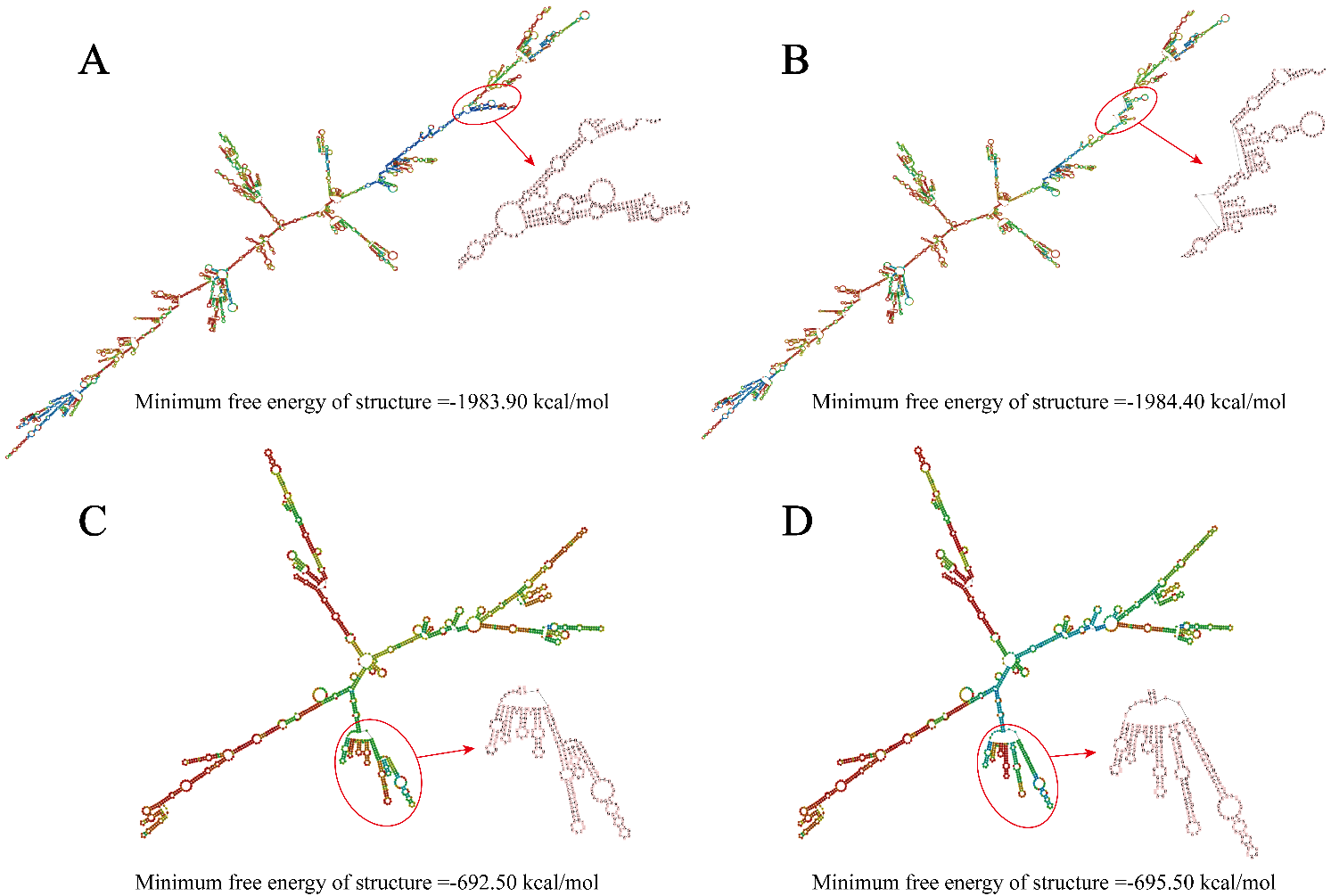


**Supplementary Figure 1.** Prediction of mRNA secondary structure before and after nsSNPs mutation. A and B were the mRNA secondary structure before and after mutation at KIAA1217 g.24429511T>C site, respectively. C and D were the mRNA secondary structure before and after SNTA1 g.62222626C>A mutation, respectively. The red part indicates the difference before and after the mutation, and the number at the bottom of the figure indicates the minimum free energy value.
